# Reaching consensus on GP interprofessional competencies: a nominal group study

**DOI:** 10.3399/BJGPO.2021.0243

**Published:** 2022-07-13

**Authors:** Stijn Duijn, Anneke van Dijk-de Vries, Nynke D Scherpbier-de Haan, Diana HJM Dolmans, Jean WM Muris, Marloes A van Bokhoven

**Affiliations:** 1 Department of Family Medicine, Maastricht University, Care and Public Health Research Institute (CAPHRI), Maastricht, The Netherlands; 2 Department of General Practice and Elderly Care Medicine, University Medical Centre Groningen, Groningen, The Netherlands; 3 Department of Educational Development & Research, Maastricht University, Faculty of Health, Medicine and Life Sciences, School of Health Professions Education, Maastricht, The Netherlands

**Keywords:** interprofessional collaboration, competency framework, workforce, nominal group, primary health care, general practitioners

## Abstract

**Background:**

As the requirements for collaboration in primary care increase, effective interprofessional teamwork between GPs and other primary care professionals is crucial. The need for more training in interprofessional collaborative competencies is widely recognised. However, existing competency frameworks do not sufficiently specify interprofessional collaboration to guide interprofessional competency development.

**Aim:**

To reach consensus among GPs and other primary care professionals on interprofessional competencies that GP and GP trainees should learn.

**Design & setting:**

A qualitative consensus study among Dutch GPs and other primary care professionals, all with expertise in primary care interprofessional collaborative practice.

**Method:**

Three nominal group sessions were held, each resulting in its own group consensus on GP interprofessional collaborative competencies. The researchers conducted a content analysis to merge and thematise the prioritised competencies into one list. Participants prioritised this list of competencies. A pre-set cut-off point was applied to determine the overall consensus on core GP interprofessional competencies.

**Results:**

Eighteen professionals from nine different disciplines participated. The content analysis resulted in 31 unique competencies, of which 14 competencies were prioritised in the final ranking into the following three main themes: (1) professional identity development and role definition by the GP (three competencies); (2) developing and executing shared care plans for individual patients (six competencies); and (3) initiating and maintaining interprofessional collaborative partnerships (five competencies).

**Conclusion:**

An interprofessional group of experts reached consensus on 14 competencies within three themes. This framework provides a stepping stone for GPs to focus on their development regarding interprofessional collaboration.

## How this fits in

The need for more training in interprofessional collaborative competencies is widely recognised. Existing collaboration competency frameworks either focus on competencies that are applicable to all healthcare professionals, regardless of their discipline, or specifically on GP competencies with limited attention to interprofessional teamwork. The framework presented in this article integrates both perspectives. It shows which competencies a GP trainee should develop to start working as a competent professional in collaborative care practice in primary care.

## Introduction

In the 21st century, the core values of general practice are to provide person-centred, holistic, and comprehensive care.^1,2^ GPs face an increase in complexity of care demand in their practices due to substitution of care from secondary to primary care, and a growing ageing population. Providing longitudinal, comprehensive patient care has become a matter of teamwork with professionals from various backgrounds and with complementary roles in the team.^3^

In complex care settings, a care approach in which different healthcare professionals provide care in an independent and sequential way is not adequate.^4^ Instead, interprofessional care, defined as *'multiple health professionals from different professional backgrounds* [working] *together with patients, families, carers, and communities to deliver the highest quality of care'* ^5^ is recommended by the World Health Organization (WHO) and others.^5–8^ With the implementation of new ways for collaborative practice, programmes need to pay attention to the interprofessional relationships between all healthcare providers to fulfil their maximum potential.^9,10^

Just like every other profession in primary care, GPs have a specific role within interprofessional collaboration. Both GPs and other primary care professionals allocate the GP a central role in collaboration within primary care.^11,12^ As well as being a medical expert, the GP is the constant factor in longitudinal care for an individual patient; they are equipped to take a ‘helicopter view’, and often coordinates care.^11,12^

GPs and GP trainees both express the need for more learning and development of interprofessional collaborative competencies.^12–14^ Therefore, the following question arises: which competencies should be acquired by GP trainees to fulfil their role in interprofessional collaboration in primary care?

In GP-specific competency frameworks from The Netherlands, UK, Australia, and Canada, interprofessional competencies have not been specified.^15–18^ The frameworks are mainly composed by and for physicians, and therefore describe collaboration from the uni-professional perspective of the GP or only describe those competencies that are applicable to all collaborative partners and not specific for GPs or GPs in training.^7,19,20^

There is no framework describing the interprofessional competencies for GPs specifically. The aim of this study is therefore to develop a competency framework for GPs with regard to interprofessional collaboration within primary care, based on consensus between GPs and other primary care professionals.

## Method

### Study design

**Figure 1.**
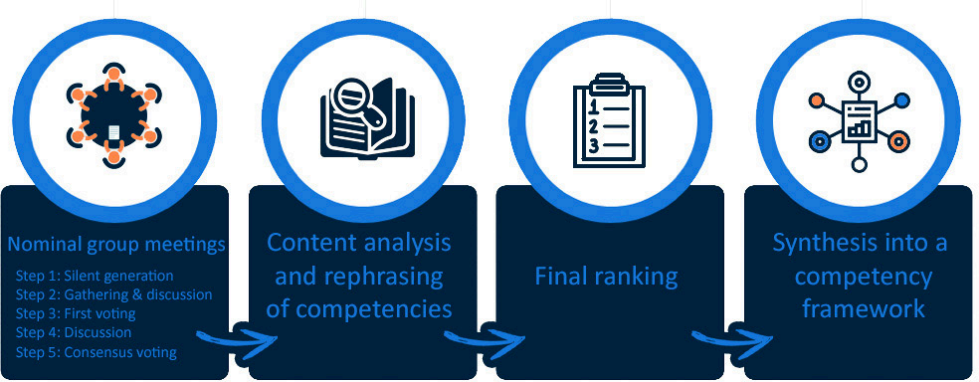
Schematic overview of the study design

Nominal group technique (NGT) was used to reach consensus about competencies of GPs regarding interprofessional collaboration with primary care health professionals. NGT is a structured method for generating a group consensus with equal contribution of every participant.^21^ Qualitative idea generation and group discussion are integral parts of NGT. It enables consensus-building based on ideas from different perspectives, an interprofessional discussion, and equal input and voting rights for all participants. The NGT, as described by McMillan *et al,* was modified to facilitate multiple separate groups by adding a content analysis and a final ranking among all participants (see Figure 1).^21^

### Participant sampling

Three group meetings were organised to include a heterogeneous sample of primary care health professionals from different regions in The Netherlands (Maastricht, Nijmegen, and Utrecht).

To gain input from people with a broad expertise in learning interprofessional collaboration competencies, the research team purposefully invited healthcare professionals who combined their daily clinical practice with a function as teacher, interprofessional care researcher, or policymaker. The aim was to invite a minimum of seven experts per group, including at least two experts with a background as a GP. Given the focus on interprofessional competencies, the other participants came from a broad variety of professional backgrounds in primary care, including a psychologist, physiotherapist, dietician, occupational therapist, district nurse, pharmacist, and social worker (see Table 1). Participants were invited via email. Participation in this study was voluntary. Written informed consent was obtained from all participants. All data were anonymised and stored on an encrypted server at Maastricht University. There was a monetary reward of €25 in gift cards for all participants.

**Table 1.** Participant characteristics and group characteristics

Professional background	Group 1 (*n* = 7)		Group 2 (*n* = 6)		Group 3 (*n* = 5)	
GP	2		2		2	
Psychologist	2		–		2	
Physiotherapist	1		1		–	
Dietician	1		–		–	
Occupational therapist	1		–		–	
District nurse	–		1		–	
Pharmacist	–		1		–	
Social worker	–		1		–	
Educationalist	–		–		1	
Sex (# female)	5		5		2	
	*Mean*	*Range*	*Mean*	*Range*	*Mean*	*Range*
Age in years	46.71	35–56	50.00	33–62	52.75	45–64
Years in practice	22.00	6–33	19.00	10–32	22.75	6–38
Main professional activities^a^						
Patient care	4		6		2	
Education	2		1		1	
Research	1		–		–	
Policymaking	–		1		4	

^a^Some participants stated two main activities

### Data collection

#### Nominal group meetings

The NGT facilitated the development of a bottom-up consensus about a framework of competencies. Three nominal group meetings were organised from March–May 2019. The duration of the group meetings ranged from 105–141 minutes. One independent moderator, with a background as a GP and educationalist, chaired all groups. Two researchers were present to assist the group discussion and to take field notes. The group meetings were audio-recorded and transcribed verbatim.

The meetings followed a standardised procedure, according to McMillan *et al* (see Figure 1).^21^ One of the researchers (SD, a GP trainee and PhD student) started with an introduction to ensure mutual understanding of the definitions of competencies and the NGT procedure. Then, participants were asked the following question: what are the crucial competencies for a GP regarding interprofessional collaboration within primary care? Each participant generated a list of competencies in silence (step 1).

Next, the participants compiled a preliminary list of competencies by taking turns stating one of their written competencies at a time. This continued until all participants had stated all of their written-down competencies.

The group discussed this list and could decide to change or merge competencies (step 2). After the discussion, participants each ranked the five most important competencies in a vote, from one to five with the highest number valued the most (step 3). Qualtrics (https://www.qualtrics.com) was used to vote and tally. The results of the first voting round were presented on screen.

A second discussion and consensus voting round followed in the same way as the first one, but now the participants voted on all competencies that achieved at least one point in the first round (steps 4 and 5). This voting round resulted in an individual group consensus for each group. All competencies that received at least one vote during the last voting round of the NGT sessions were taken into account in the content analysis.

#### Content analysis

The three nominal group sessions were followed by a content analysis of the three individual group consensus statements by three researchers: SD, AvD (a post-doctoral researcher and health scientist), and MvB (an associate professor and GP). First, two researchers (SD, AvD) analysed the group consensuses and transcripts independently from each other. During this phase, it became clear that the third nominal group meeting did not reveal any new major themes, so no additional meetings were needed. Second, three researchers merged competencies that were mentioned more than once. Other competencies were rephrased into single measurable behaviours. Some competencies contained multiple elements and were therefore split into two or three competencies. This resulted in 31 unique competencies.

The researchers grouped the competencies into three emerging main themes, to facilitate reading and voting. Transcripts of the meetings helped to understand the context and formulation of different competencies. After the first analysis, the three researchers themed the competencies into a voting longlist. The entire research group finalised the thematisation and the longlist for ranking.

#### Final ranking

The longlist was sent to all participants for a final round of ranking, using Qualtrics. The order in which competencies were presented was randomised for each participant. Using the 100-points method, participants were asked to distribute 100 points freely among all competencies of each of the three themes.

#### Synthesis into a competency framework

Given the goal of developing a comprehensive competency framework that is feasible at the workplace, the authors wanted to include 4–6 competencies for each theme. Therefore, a cut-off was chosen of at least 10% of the available points within a theme in advance, to include an individual competency in the framework. This percentage was chosen as it resembles the mean score when all 31 competencies resulting from the analysis would receive an equal number of points.

## Results

### Nominal group sessions

Eighteen professionals participated in the group sessions (5–7 per group). Characteristics of the three different groups are summarised in Table 1. Groups one, two, and three, respectively, generated a list of 12, 8, and 8 competencies in the consensus voting round (step 5).

### Content analysis

During the subsequent content analysis of the 28 competencies from the NGT sessions, 15 competencies were merged, six competencies were split into two competencies, and two competencies were split into three competencies. During the content analysis, the researchers grouped the prioritised competencies into three themes (see Table 2). The analysis resulted in a voting list for the final ranking, consisting of three themes with in total 31 competencies (9, 11, and 11 competencies allotted to each theme, respectively).

**Table 2.** Final ranking

Themes and competencies	Points (%)^a^
Theme 1: Professional identity development and role definition by the GP	
Knows the expertise, tasks, and work processes, and with that the (im)possibilities of collaborative partners, and can use this knowledge in daily care practice. Knows their own expertise, tasks, and work processes and can use this knowledge in daily care practice, and with that is conscious of own possibilities and boundaries. Shows awareness of the importance of interprofessional collaboration and is prepared to collaborate interprofessionally.	300 (17.7%)260 (15.3%)225 (13.2%)
Theme 2: Developing and executing shared care plans for individual patients	
Makes shared decisions with patients, patients' families and carers, and healthcare professionals. Is available for consultation, knows how to make priorities and to set boundaries. Sees who can take the lead in a care plan and dares to delegate. Informs collaborative partners proactively and on time. Recognises and uses the possibilities of collaboration in the problem analysis. Knows the social network and context of the patient.	320 (18.8%)235 (13.8%)185 (10.9%)185 (10.9%)185 (10.9%)180 (10.6%)
Theme 3: Initiating and maintaining interprofessional collaborative partnerships	
Works out agreements with collaborative partners regarding roles, care goals, responsibilities, possibilities for up-scaling, and feedback moments. Develops a shared vision with other primary care professionals regarding the collaborative partnership. Initiates and maintains collaborative relationships with individual collaborative partners as well as within an interprofessional collaborative network proactively. Evaluates the agreements and the collaboration itself with collaborative partners. Shows leadership, expressed by decisiveness, direction, inspiration, creativity, delegation, negotiation, maintaining structure, conflict management, and overseeing progress.	445 (26.2%)310 (18.2%)210 (12.4%)175 (10.3%)175 (10.3%)

^a^Cumulative number of allocated points by all participants (% of total allocated points in each theme).

### Final ranking

Response rate on the final ranking round was 17/18 participants. In the first theme, 3 out of 6 competencies reached the cut-off; in the second theme 6 out of 11 competencies; and in the third theme, 5 out of 11 competencies. The themes and competencies that reached the cut-off are presented in Table 2. The full table with all competencies included in the list sent to participants is presented in Supplementary appendix 1.

### Themes

The three themes that emerged from the content analysis were as follows: (1) professional identity development and role definition by the GP; (2) developing and executing shared care plans for individual patients; and (3) initiating and maintaining interprofessional collaborative partnerships. The following are the characteristics of these themes.

#### Professional identity development and role definition by the GP

The first theme is about the GP developing a professional identity and role definition. Competencies within this theme revolved around GPs knowing their own expertise and processes, what their role is in collaboration, and what they would like to achieve for their patients. Important in developing their own identity and role definition is comparing themselves with other professionals in their health landscape. Another competency in this theme, therefore, is knowing the professionals who could provide care for shared patients, knowing what to expect from them, including what they could contribute to care, and their boundaries of expertise and responsibilities.

#### Developing and executing shared care plans for individual patients

The second theme is about providing collaborative care to the individual patient. First, the experts agreed that a GP needs to be able to recognise situations where collaboration can be an asset, and to initiate the collaboration with the right other primary care professionals. The GP should, in conjunction with the patient, their families, carers, and other necessary healthcare professionals, facilitate the team to set joint care goals. When setting the care goals, the GP adds unique value through the longitudinal aspect of care provided and their knowledge about the patient and their context over time. The experts agreed that GPs should proactively inform other collaborating healthcare professionals about new developments while care is ongoing. This was pointed out by the experts despite medical care ultimately being the GP’s responsibility. The GP needs to be open for other healthcare professionals who might be better equipped in taking the lead in some care situations. This requires trust and to be consulted by the other professionals when necessary.

#### Initiating and maintaining interprofessional collaborative partnerships

The third theme is about initiating and maintaining collaborative partnerships with professionals from other primary care disciplines by the GP. According to the expert group, GPs need to be able to enter into agreements with other primary care professionals regarding roles, responsibilities, possibilities for upscaling care, and feedback moments. The experts emphasised the importance of GPs building a network of other primary care professionals, and maintaining this network proactively. In arranging this network with other primary care professionals, a GP should bring parties together to develop a clear collective vision on the care that they want to provide as an interprofessional team. The experts would prefer that the GP guides the group, for example, by being inspiring and creative, because they view the GP as the natural leader. Within this leadership role, the GP should direct, negotiate, delegate, and resolve any conflicts. When needed, a GP should be decisive and helpful in maintaining structure in meetings. The GP is best suited to oversee the group as well as to oversee the care process as a whole. GPs should thus be able to initiate collective evaluation moments, where feedback from and to all members is possible, with the aim of providing even better care.

## Discussion

### Summary

An interprofessional panel of primary care professionals with expertise in interprofessional collaboration reached consensus on 14 core competencies for GP interprofessional collaboration. These core competencies are divided into the following three main themes: (1) professional identity development and role definition by the GP; (2) developing and executing shared care plans for individual patients; and (3) initiating and maintaining interprofessional collaborative partnerships.

### Strengths and limitations

This study has strengths and weaknesses. A strength is that the developed framework not only describes generic interprofessional competencies but also the competencies required for the unique role of the GP in an interprofessional setting. The sample and methodology provided a sound basis to define the significant interprofessional collaborative competencies of GPs. A review on the NGT stated that samples between two and 14 participants have been used and a number of about seven participants is recommended.^21^ To improve robustness, the NGT procedure was repeated in three different regions of the country. Moreover, the environment was not limited to GPs alone but included primary care collaborating professionals from other disciplines as well. To avoid misinterpretations, a member check was done among all participants. It should be noted that the study was in the context of Dutch primary care, which does not necessarily mean that it is applicable everywhere. However, in comparable primary care settings, like in many countries in Europe, the results could be applied.

The exclusion of patients may have resulted in insufficient attention to person-centredness of care. However, theme 2, on developing shared care plans, explicitly mentioned patient-centredness in shared decisionmaking and knowledge of the patient’s context. The research team also chose to exclude GP trainees, which could mean that the framework is less applicable in the setting of GP training, though GP supervisors and educators were represented, and GP trainees may have a blind spot regarding the required competencies. Another limitation of this study is that a consensus procedure by voting requires authors to pre-define an arbitrary 10% cut-off. However, after voting, this cut-off did indeed lead to the intended manageable number of competencies per theme.

### Comparison with existing literature

Professionals in primary care need both generic competencies (applicable to all health professionals) and profession-specific competencies (to fulfil the unique role of their discipline). Existing competency frameworks do not sufficiently cover generic and profession-specific interprofessional collaborative competencies. GP frameworks have described GP-specific competencies, but do not provide much detail regarding the generic interprofessional competencies such as knowing your own expertise or being available for consultations.^15–18^ On the other hand, interprofessional frameworks have described the generic interprofessional competencies, but do not give attention to profession-specific competencies.^7,19,20^ The framework, as presented in this study, appears to cover both types of competencies.

The leadership role that was assigned to GPs in interprofessional teamwork may be misinterpreted as GPs being seen as superior to other team members. According to the present study's competency framework, GPs needed to both be able to see who can take the lead in an individual care plan, and to delegate. This is in line with Varpio and Teunissen, who argued that all members of an interprofessional healthcare team need to be able to act as both leaders and followers, changing roles as the situation requires.^22^ Furthermore, the participants in the present study, both GPs and other primary care professionals, prioritised initiating and maintaining collaborative partnerships (theme 3) as a GP interprofessional competency. Other studies have also concluded that primary care professionals attribute this kind of leadership role to GPs in the care of frail older people. They have stated that GPs are medical experts who are a constant factor in care. Furthermore, the studies found that GPs are equipped to ‘*see the bigger picture*’ and are capable of networking with relevant partners at a strategic level.^11,23^ Whether other professionals, who have settled in a community for a long time and see the bigger health picture, could perform this role as well is beyond the scope of the present study.

The framework in the present study showed which competencies a GP should develop to start working as a competent professional in collaborative care practice in primary care. One may question where GPs need to develop these competencies: do they need an interprofessional practice-based setting? Paradis and Whitehead argued that not all education regarding interprofessional care practice should take place in interaction with other professionals.^24^ In the present study's framework, this could be the case for some of the interprofessional competencies in theme 1, mainly regarding GP trainees getting to know their own roles and the tasks of other health professionals. Frenk *et al* argued, however, that collaboration in a workplace setting is always necessary in interprofessional competency development.^25^ This may be the case for a number of competencies in the framework; for example, the competencies of theme 2 regarding direct collaborative patient care. Improving these elements of interprofessional teamwork is a cyclic process and therefore needs to take place in practice.^25,26^ The focus in theme 3 is on long-term collaboration. Long-term collaborative partnerships require trust and shared experiences among different professionals that take time to develop.^27–29^ Full development of theme 3 could starts during GP training, but it calls for a lifelong-learning process.

The three themes of the framework may have suggested that there is a specific order in the development of all competencies. There is no clear answer to this. Research by van Dongen *et al* suggested there is at least some hierarchy in developing interprofessional competencies since knowing yourself and the expertise of other professionals has been put forward as a requirement to engage in interprofessional practice. Besides, it underlines that long-term collaboration requires trust and shared experiences.^30^

In addition to the issues regarding where and when to be trained in interprofessional practice, one may also question how GPs could develop their interprofessional collaboration competencies. Previous studies provide some suggestions. For example, Elwyn *et al* described the three-talk model as a useful guide for interprofessional shared decisionmaking.^31^ The framework, as described by van Dongen *et al*, facilitates a team evaluation in order to improve long-term collaboration.^26,27^ The latter may be helpful for the competencies in the third theme. However, for most of the competencies of the present framework, the question on how to develop the competencies is still unanswered.

### Implications for research and practice

This study has provided a deeper understanding of what GPs should learn to become effective interprofessional collaborators in primary care. The training of GP trainees consists largely of working in clinical practice, which is an effective learning environment. This particularly applies to developing competencies with regard to interprofessional teamwork. However, explicit attention to the development of collaborative skills is not self-evident, since the focus of GP trainees is on their clinical tasks and communication skills in patient consultations. The framework presented in this study is intended to be used by GP trainees to set learning goals that help them reflect on and shape their further professional development with regard to collaboration. This appeals to supervisors to create explicit interprofessional learning situations at the workplace. A question for further research is what both GP and GP trainees need to facilitate the GP trainees’ learning of interprofessional competencies at the workplace.

Research has suggested that GP trainees should be challenged to seek reliable and valid external feedback in addition to their self-assessment to enrich the learning process.^32^ This raises the question of whether other primary care professionals could play a role in the learning process of the GP trainee. If yes, what would they need, in order to provide meaningful feedback to GP trainees regarding their interprofessional competencies? This is also a topic for further research.

## Data

The data that support the findings of this study are available from the corresponding author upon reasonable request.
